# Efficacy of escitalopram for poststroke depression: a systematic review and meta-analysis

**DOI:** 10.1038/s41598-022-05560-w

**Published:** 2022-02-28

**Authors:** Rong-fang Feng, Rui Ma, Peng Wang, Xu Ji, Zhen-xiang Zhang, Meng-meng Li, Jia-wei Jiao, Li Guo

**Affiliations:** 1 The First Affiliated Hospital of Zhengzhou University, Zhengzhou, 450052 Henan People’s Republic of China; 2 College of Physical Education (Based School), Zhengzhou University, Zhengzhou, 450001 Henan People’s Republic of China; 3 Department of Basic Medicine, School of Nursing and Health, Zhengzhou University, Zhengzhou, 450001 Henan People’s Republic of China; 4 Zhengzhou University of Industrial Technology, Zhengzhou, 450002 Henan People’s Republic of China; 5 Medical School of Huanghe Science and Technology University, Zhengzhou, 450006 Henan People’s Republic of China; 6 Henan University of Chinese Medicine, Zhengzhou, 450046 Henan People’s Republic of China; 7 Department of Clinical Medicine, School of Nursing and Health, Zhengzhou University, Zhengzhou, 450001 Henan People’s Republic of China; 8 Zhengzhou Central Hospital Affiliated to Zhengzhou University, Zhengzhou, 450007 People’s Republic of China

**Keywords:** Diseases, Health care, Neurology

## Abstract

Depression is very common after stroke, causing multiple sequelae. We aimed to explore the efficacy of escitalopram for poststroke depression (PSD). PubMed, Embase, Scopus, Cochrane Central Register of Controlled Trials, Clinical trials. gov, Wan fang Data (Chinese), VIP (Chinese) and CNKI (Chinese) were retrieved from inception to May 2021. We recruited Randomized Controlled Trials (RCTs) which met the inclusion criteria in our study. The depression rating scores, the incidence of PSD, adverse events as well as functional outcomes were analyzed. 11 studies and 1374 participants were recruited in our work. The results were depicted: the reduction of depression rating scores was significant in the escitalopram groups and the standard mean difference (SMD) was − 1.25 (*P* < 0.001), 95% confidence interval (95% CI), − 1.82 to − 0.68; the risk ratio (RR) of the incidence of PSD was 0.52 (95% CI, 0.29 to 0.91; *P* = 0.007 < 0.05), which was significantly lower in the escitalopram groups; Escitalopram is safe for stroke patients; there was improvement of the motor function. However, in sensitivity analyses, the conclusions of the motor function and the incidence of drowsiness were altered. The study suggests that escitalopram has a potentially effective role compared with control groups and demonstrates escitalopram is safe. However, the results of the motor function and the incidence of drowsiness should be considered carefully and remain to be discussed in the future.

## Introduction

Approximately 79,5000 people suffer a new or recurrent stroke each year^1^. Additionally, an epidemiology meta-analysis revealed 31% of patients developed depression during 5 years following stroke^2^. Frustratingly, poststroke depression (PSD) could impair the cognitive level and activities of daily living (ADL), cause negative sequelae on the recovery of patients, and increase the burden of caregivers^3^.

So far, the etiological mechanisms of PSD have not been revealed clearly. Psychological, social, and biological factors contributed to PSD together^4,5^. Stroke survivors with the homozygous short variation allele genotype of the serotonin transporter-linked polymorphic region (5-HTTLPR) have a higher risk of PSD^6^. Both stroke and depression are associated with increased inflammation^7^. Antidepressants can lower the levels of pro-inflammatory cytokines^8^. These new promising methods show that, in terms of the physical consequences of stroke, these drugs can reduce bad mood^9^. Escitalopram is a selective serotonin reuptake inhibitor (SSRI) with few drug interactions, and is thus suitable for stroke patients who are prescribed multiple medications^10^. In recent years, SSRI escitalopram has been proved to be effective for the treatment and prevention of PSD, but there are still controversy^11,12^.

It demonstrates that escitalopram is safe in a randomized controlled trial (RCT) for prevention of PSD, and decreases effectively the incidence of PSD, as well as improving ADL and social function^11^. Moreover, it also shows that there are no significant differences on cognitive function compared with problem-solving therapy (PST) and placebo. However, a study of escitalopram by Kim et al.^12^ shows that the occurrence of moderate or severe depressive symptoms and adverse events are not statistically significant except diarrhea, ADL improvement, cognitive function, motor function and neurological defects.

Two SSRI systematic reviews^13,14^ enrolled RCTs of escitalopram have been found, to our knowledge, which both only included a study of Robinson et al.^11^. Recently, new RCTs of escitalopram are pouring out^15–23^, which were conducted in different circumstances, and the integration effects of these studies was vague.

Therefore, we aimed at conducting a systematic review and meta-analysis of RCTs about escitalopram arm compared with the placebo/the blank control arm, evaluating the depression rating scores, the occurrence of depression along with depressive symptoms, the frequency of adverse events and other significant clinical outcomes.

## Methods

### Search strategy and study selection

8 databases were searched (search strategy in online supplemental data), Medline via PubMed, Embase, Scopus, Cochrane Central Register of Controlled Trials, Clinical Trials.gov, CNKI (Chinese), Wan fang (Chinese), and VIP database (Chinese), from inception to May 2021. In addition, we scrutinized references of relevant papers and also contacted with authors to get the detailed data if necessary.

Inclusion criteria: ① RCTs were enrolled for participants with a clinical diagnosis of stroke; ② The experimental group was treated with escitalopram at any dose, by any mode of delivery and the control arm was included a placebo or the blank control; ③ The primary outcomes: depression rating scores, in which the Hamilton Depression Scale (HAMD) was preferred, the incidence of PSD, and adverse events including gastrointestinal side effects, sexual side events, cardiovascular adverse effects, and other adverse events. The secondary outcomes: neurological deficit scores, ADL, cognitive impairments, and motor function. For functional outcomes, we gave preference to the National Institutes of Health Stroke Scale (NIHSS), the Barthel Index (BI), Fugl—Meyer motor scale (FM), Mini-Mental State Examination (MMSE) and Montreal Cognitive Assessment (MoCA).

Exclusion criteria: ① The type of study was a non-randomized controlled study; ② The subjects were not stroke patients or no clear diagnostic criteria; ③ The experimental group was not treated with escitalopram, or the control arm was not included a placebo or the blank control, or drugs and therapies with mixed effects; ④ Outcome indicators were not required in this study; ⑤ Intervention methods were not expressed clearly and could not be verified by the authors.

Two team members exacted data of each literature independently. A third investigator was discussed with if necessary.

### Quality assessment

Study quality was independently assessed by two reviewers based on the Cochrane Collaboration’s risk of bias tool including randomization, allocation concealment, blinding, incomplete outcome data, and selective reporting. An opinion was sought from a third reviewer if the first two reviewers could not reach an agreement.

### Statistical analysis

Pooled analyses were carried out at any follow-up point by RevMan 5.3 software (Copenhagen: The Nordic Cochrane Center, The Cochrane Collaboration, 2014). Risk ratio (RR) with 95% confidence interval (CI) was described by categorical data. Standardized mean difference (SMD) with 95% CI was used for continuous outcomes. *P* < 0.05 was used as a cutoff for statistical significance. Statistical heterogeneity of trials was evaluated by I^2^^24^. We used a random-effect model to calculate the pooled estimates if we observed I^2^ > 50% or *P* < 0.10, and on the contrary, we used a fixed-effect model.

Subgroup analyses were conducted based on different rating scales, depression or not at recruitment and follow-up duration (< 3 months vs 3 ~ 6 months vs > 6 months).

In sensitivity analyses, the trails with high heterogeneity were excluded. Publication bias was assessed by a funnel plot and Egger statistical test that was carried out by Stata 12.0 and *P* < 0.10 was considered as statistically asymmetry^25^.

Our study was conducted according to the PRISMA 2020 guidelines. We analyzed the data about previous studies which were published early in our research, so ethical approval and patient consent were not necessary and therefore not provided.

## Results

### Study selection and characteristics

**Figure 1:**
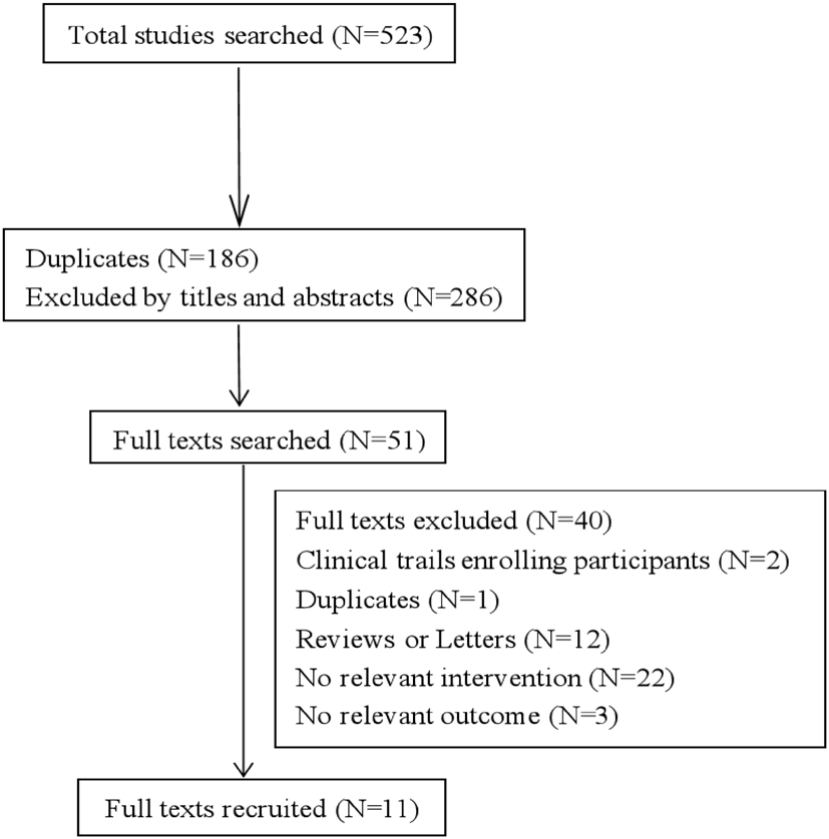
Flow diagram of the paper search.

We searched 5 English databases (27 from Medline via PubMed, 72 from Embase, 123 from Scopus, 12 from Cochrane Central Register of Controlled Trials, 3 from Clinical Trials.gov) and 3 Chinese databases (100 from CNKI, 99 from Wan fang, 87 from VIP database) from inception to May 2021. After removing duplicates, there existed 285 records and 51 full texts were obtained. Finally, 11 articles were recruited (Fig. 1), in which 1374 participants were randomly enrolled in the escitalopram or the control^11,12,15–23^. Most of the papers excluded the participants with severe comprehension deficits, aphasia, and unstable medical conditions.

The follow-up was at treatment end in 9 RCTs^11,12,15,17–23^. There were 2 RCTs of which the follow-up duration was beyond treatment end^12,16^, however, we could not obtain the detailed data of Kim et al.^12^ at 6 months and Mikami et al.^16^ at 18 months. Participants suffered from depression at recruitment in 5 papers^12,19,20,22,23^. In 6 papers, participants were with no diagnosis of depression at recruitment^11,15–18,21^. Table 1 shows the detailed characteristics of each paper.

**Table 1:** Characteristics of the studies recruited.

Study	Sample (n) Esci/Control	Age (SD)	Time since stroke (SD)	Diagnosis criteria	Treatment methods	Dose (mg/d)	Evaluation time point	Outcome Indexes
Robinson et al.^11^ (English)	59/58	50–90	< 3 months	Clinically documented stroke, HAMD-17 ≤ 11, No depression meeting DSM-IV criteria	Esci/Placebo	5–10	3, 6, 9, 12 months	Incidence of PSD, adverse events
Kim et al. ^12^ (English)	241/237	Esci: 63.6(12.0) Placebo: 63.5 (12.6)	< 21 days	Clinically documented stroke, modified Rankin Scale ≥ 2	Esci/Placebo	5–10	3, 6 months	NIHSS, BI, MoCA, Motor function, Adverse events
Jorge^15^ (English)	43/45	50–90	< 3 months	Clinically documented stroke, HAMD-17 ≤ 11, No depression meeting DSM-IV criteria	Esci/Placebo	5–11	3, 6, 9, 12 months	RBANS, Adverse events
Mikami et al. ^16^ (English)	34/33	50–90	< 3 months	Clinically documented stroke, No depression meeting Manual of Mental Disorders, Fourth Edition diagnosis and HAMD	Esci/Placebo	5–10	6 months	HAMD-17, Incidence of PSD, adverse events
Zhan et al.^17^ (Chinese)	40/42	Esci: 65.93(7.85) Placebo: 64.00(6.87)	5–10 days	Diagnostic criteria for the Fourth National Conference on cerebrovascular diseases in China and CT or MRI, FM < 55, NIHSS ≤ 20	Esci/Placebo	10	90 days	HAMD-17, Incidence of PSD, NIHSS, FM, Adverse events
Zhan et al.^18^ (Chinese)	36/37	Esci: 64.8(8.1) Placebo: 62.7(6.6)	5–10 days	Diagnostic criteria for the Fourth National Conference on cerebrovascular diseases in China and CT or MRI, MoCA < 26	Esci/Placebo	10	90 days	HAMD-17, Incidence of PSD, MoCA, BI
Wang^19^ (Chinese)	68/68	Esci: 63.8 (5.1) Control: 63.2 (4.8)	Esci: 5.3(3.4)/ Control:4.8(3.2) years	Diagnostic criteria for the Fourth National Conference on cerebrovascular diseases in China and CT or MRI, Depression meeting ICD-10 and HAMD-24 > 20	Esci/Blank control	10–20	2, 4, 8 weeks	HAMD-24, MESSS, TESS
Jiang et al.^20^ (Chinese)	20/20	Esci: 56.00(7.20) Control: 63.2 (4.8)	Esci: 50.43(27.63) Control: 42.73 (28.24) days	Diagnostic criteria for the Fourth National Conference on cerebrovascular diseases in China and CT or MRI, Depression meeting HAMD-17 > 21	Esci/Blank control	10–20	4 weeks	HAMD-17, FM, adverse events (NR)
Zhao et al.^21^ (Chinese)	49/49	62.45(11.98)	2 days	Clinically documented stroke and CT/MRI, NIHSS ≤ 22, HAMD-17 > 20	Esci/Blank control	5–10	1, 2 months	HAMD-17, NFI, adverse events
Li et al.^22^ (Chinese)	58/58	70.2(5.5)	3.7(1.3) days	Diagnostic criteria for the Fourth National Conference on cerebrovascular diseases in China and CT or MRI, HAMD-17 ≥ 17	Esci/Blank control	5–10	1, 3, 6 months	HAMD-17, MBI, MMSE, FM
Lin^23^ (Chinese)	36/34	Esci:70.2(7.1) Control: 68.4 (6.8)	Esci:7.0 (1.9)/ Control: 7.2 (2.1) months	Diagnostic criteria for the Fourth National Conference on cerebrovascular diseases in China and CT or MRI, HAMD-17 ≥ 18	Esci/Blank control	10	8 weeks	HAMD-17, CSS, Adverse events

*BI* Barthel Index; *CSS* Chinese Stroke Scale; *Esci* escitalopram; *FM* Fugl–Meyer motor scale; *MBI* Modified Barthel Index; *MESSS* Modified Edinburgh Scandinavia Stroke Scale; *MMSE* Mini-Mental State Examination; *MoCA*, Montreal Cognitive Assessment; *NFI* Neurologic Function Impairment; *NIHSS* National Institutes of Health Stroke Scale; *NR* not reported; *PSD*, poststroke depression; *RBANS* Repeatable Battery for the Assessment of Neuropsychological Status; *SD* standard deviation; *HAMD* Hamilton Depression Scale; *TESS* Treatment Emergent Symptom Scale.

### Depression rating scores

**Figure 2:**
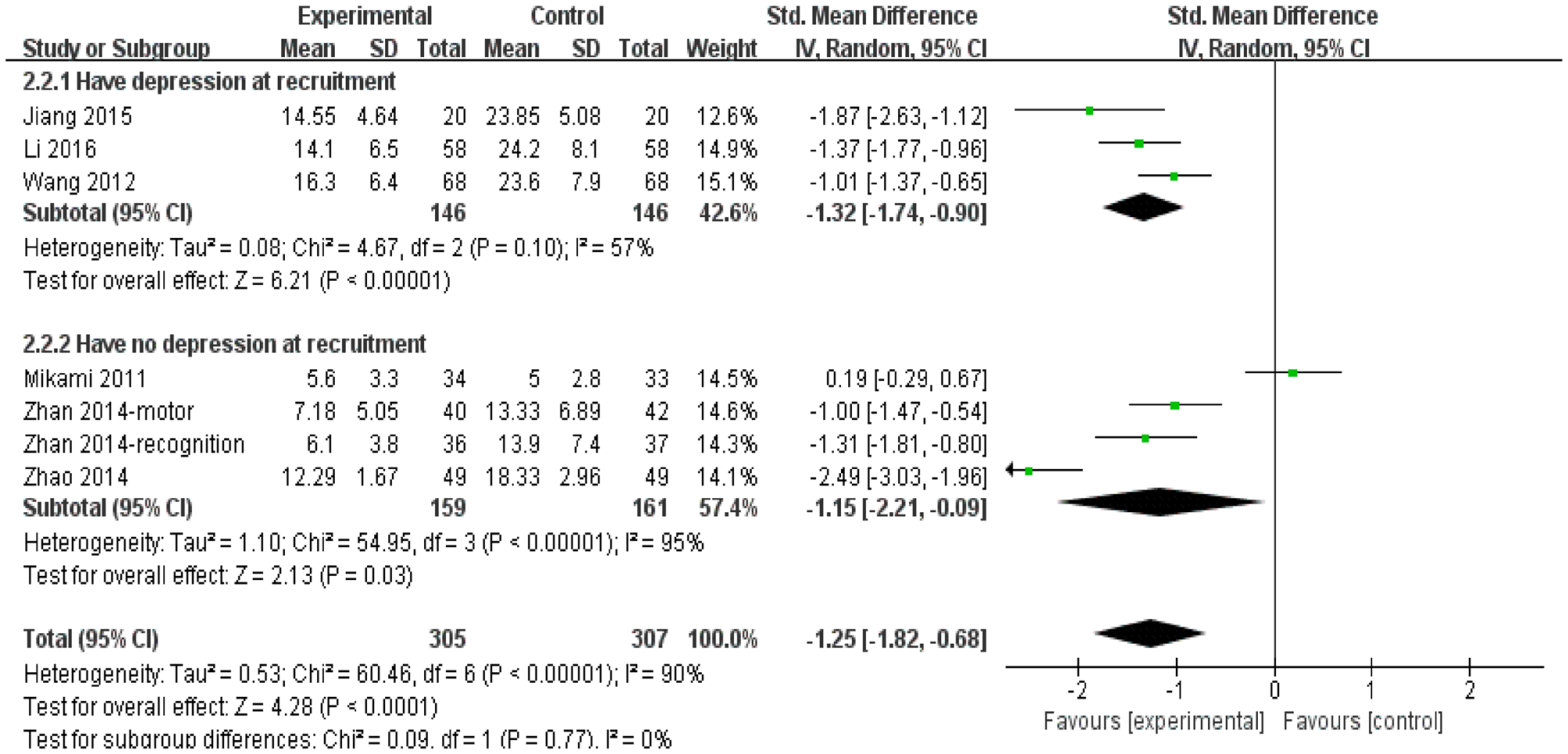
Depression rating scores of subgroup of participants with depression or not at recruitment. *CI* confidence interval.

Figure 2 shows that the SMD of depression rating scores was − 1.25 (95% CI, − 1.82 to − 0.68; 7 trials; I^2^ = 90%) among participants allocated escitalopram compared with control. But there was moderate heterogeneity among participants who were with depression (SMD = -1.32; 95% CI, − 1.74 to − 0.90; I^2^ = 57%) or not depression (SMD = -1.15; 95% CI, − 2.21 to − 0.09; I^2^ = 95%) at recruitment and with no heterogeneity between subgroups (I^2^ = 0%; *P* = 0.77). It was reported that the antidepressant efficiency was obvious statistical significance (*P* < 0.05) in escitalopram group (88.9%) compared with the control (64.7%) in one trail, but we could not obtain the detailed scores^23^.

**Figure 3:**
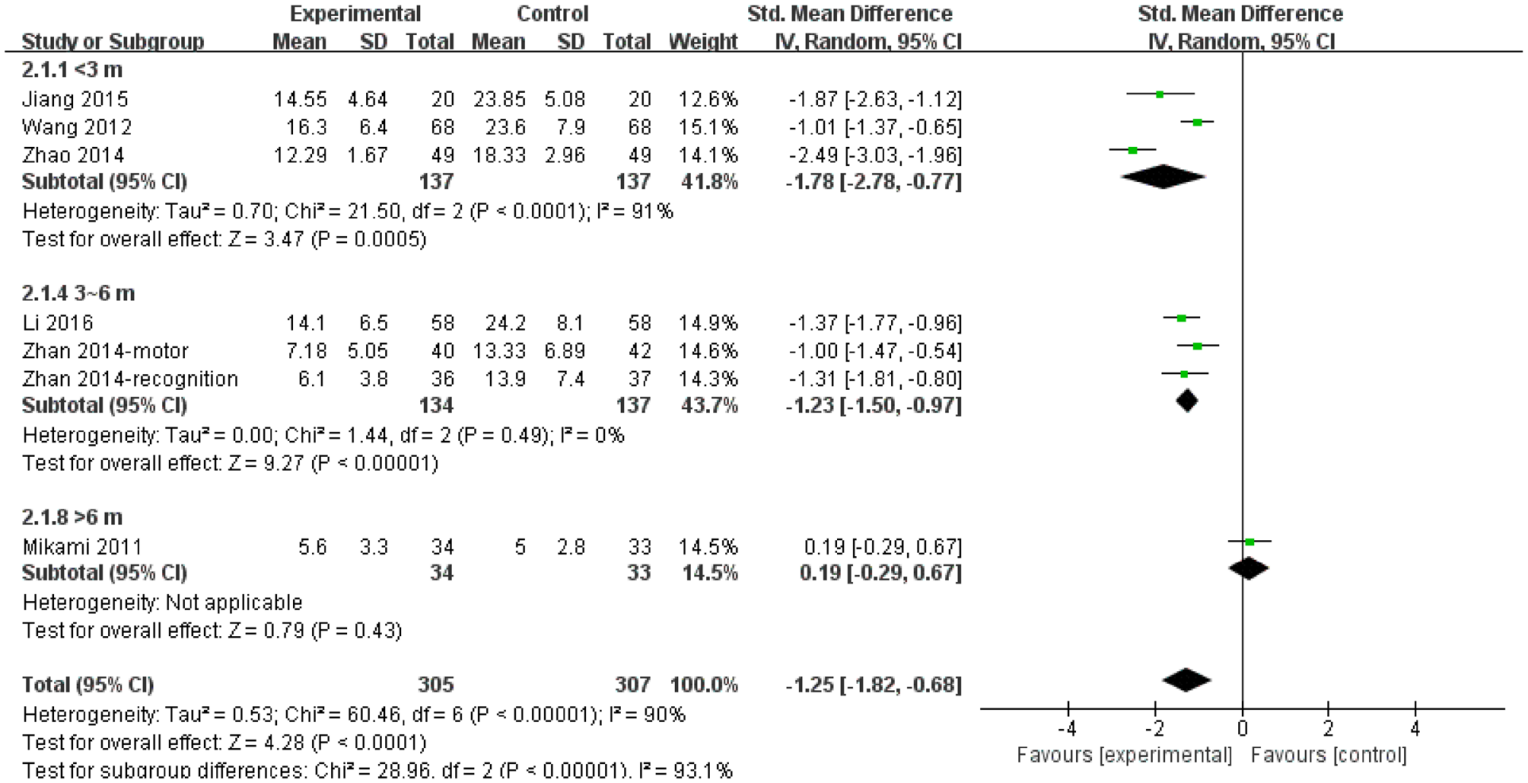
Depression rating scores of subgroup of follow-up duration (< 3 months vs 3 ~ 6 months vs > 6 months). *CI* confidence interval.

Figure 3 shows that there was obvious statistical significance in the subgroup where follow-up duration was the group of < 3 months (SMD = -1.78; 95% CI, − 2.78 to − 0.77; I^2^ = 91%) and the group of 3 ~ 6 months (SMD = -1.23; 95% CI, − 1.50 to − 0.97; I^2^ = 0%), however, there were no advantage of escitalopram in the subgroups, follow-up duration ≥ 6 months, but only one trial was included*.* Significant heterogeneity was among subgroups (I^2^ = 93.1%; *P* < 0.001).

### The incidence of poststroke depression

**Figure 4:**
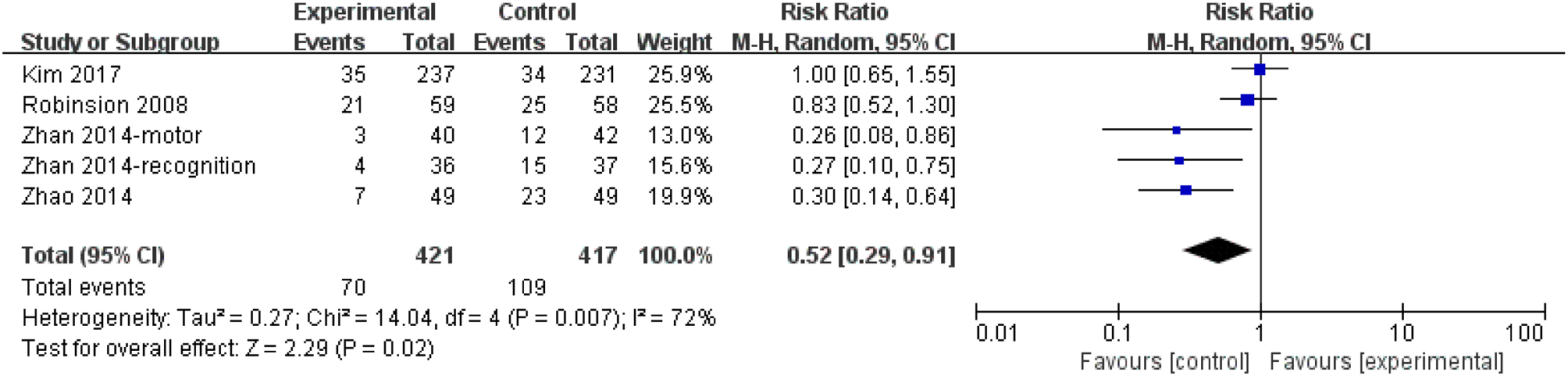
Incidence of PSD. *CI* confidence interval; *PSD* poststroke depression.

The incidence of PSD was higher in control compared with escitalopram and with moderate heterogeneity (RR = 0.52; 95% CI, 0.29 to 0.91; 5 trials; I^2^ = 72%; Fig. 4).

### Safety

**Figure 5:**
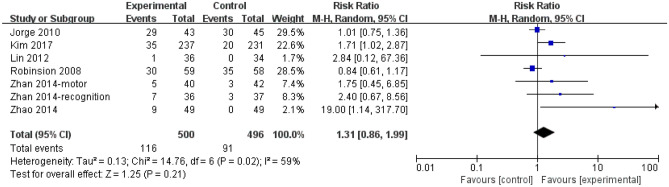
Incidence of the nausea, diarrhea, abdominal pain and constipation adverse events. *CI* confidence interval.

No statistical significance was between escitalopram and control among trials for gastrointestinal side events. For nausea, diarrhea, abdominal pain and constipation, the RR was 1.31 (95% CI, 0.86 to 1.99; 7 trials; Fig. 5) with moderate heterogeneity (I^2^ = 59%, *P* = 0.02) among trials. There was also no statistical significance for other gastrointestinal side events: the dry mouth (RR = 0.73; 95% CI, 0.52 to 1.03; 3 trials; I^2^ = 46%; Supplemental Fig. 1), the anorexia (RR = 1.66; 95% CI, 0.95 to 2.90; 3 trials; I^2^ = 2%; Supplemental Fig. 2), the indigestion (RR = 1.26; 95% CI, 0.75 to 2.11; 3 trials; I^2^ = 0%; Supplemental Fig. 3), the bleeding (RR = 1.02; 95% CI, 0.15 to 7.07; 2 trials; I^2^ = 0%; Supplemental Fig. 4).

**Figure 6:**
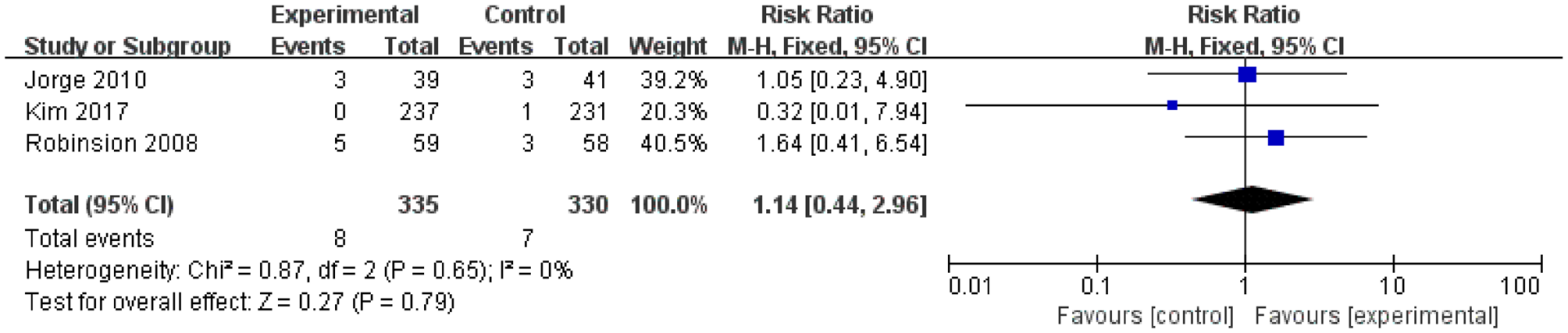
Incidence of the palpitation. *CI* confidence interval.

There was no significant cardiovascular adverse effects in escitalopram groups. The RR was 1.14 (95% CI, 0.44 to 2.96; 3 trials; I^2^ = 0%, *P* = 0.65; Fig. 6) for palpitation. For tachycardia, the RR was 1.07 (95% CI, 0.90 to 1.28; 2 trials; Supplemental Fig. 5) without heterogeneity (I^2^ = 0%; *P* = 0.65) among trials. Only 2 trails reported the chest pain, and the RR was 1.35 (95% CI, 0.68 to 2.70; 2 trials; I^2^ = 0%, *P* = 0.57; Supplemental Fig. 6).

**Figure 7:**
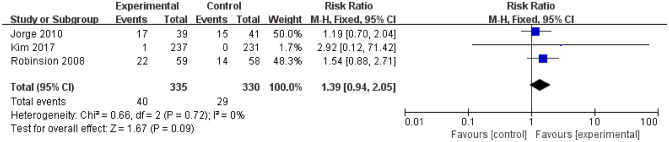
Incidence of the sexual adverse events. *CI* confidence interval.

Escitalopram did not affect sexual function versus control (RR = 1.39; 95% CI, 0.94 2.05; I^2^ = 0%, *P* = 0.72; 3 trials; Fig. 7) among trials for sexual side events.

The escitalopram was safe for the other adverse events, except for the drowsiness (RR = 6.95; 95% CI, 1.61 to 30.09; 3 trials; I^2^ = 31%, *P* = 0.23; Supplemental Fig. 7), and there was no or low heterogeneity among all enrolled trials: the insomnia (RR = 0.82; 95% CI, 0.48 to 1.39; 4 trials; I^2^ = 0%, *P* = 0.71; Supplemental Fig. 8), the dizziness (RR = 1.09; 95% CI, 0.90 to 1.32; 3 trials; I^2^ = 0%, *P* = 0.95; Supplemental Fig. 9), the fatigue (RR = 1.25; 95% CI, 0.90 to 1.74; 3 trials; I^2^ = 0%, *P* = 0.73; Supplemental Fig. 10), the increased sweating (RR = 1.78; 95% CI, 0.99 to 3.20; 3 trials; I^2^ = 0%, *P* = 0.80; Supplemental Fig. 11), the falls (RR = 1.02; 95% CI, 0.15 to 7.07; 2 trials; I^2^ = 0%, *P* = 0.97; Supplemental Fig. 12), the pain (RR = 0.88; 95% CI, 0.48 to 1.63; 2 trials; I^2^ = 24%, *P* = 0.25; Supplemental Fig. 13), the dysuria (RR = 1.38; 95% CI, 0.51 to 3.77; 2 trials; I^2^ = 0%, *P* = 0.85; Supplemental Fig. 14), the anxiety (RR = 1.98; 95% CI, 0.37 to 10.61; 2 trials; I^2^ = 48%, *P* = 0.16; Supplemental Fig. 15). It was no statistical significance (*P* > 0.05) in escitalopram group compared with the control, including the incidence of the paraesthesia, tremor, pruritus and peripheral oedema, however, they were only reported in one trail^12^.

**Figure 8:**
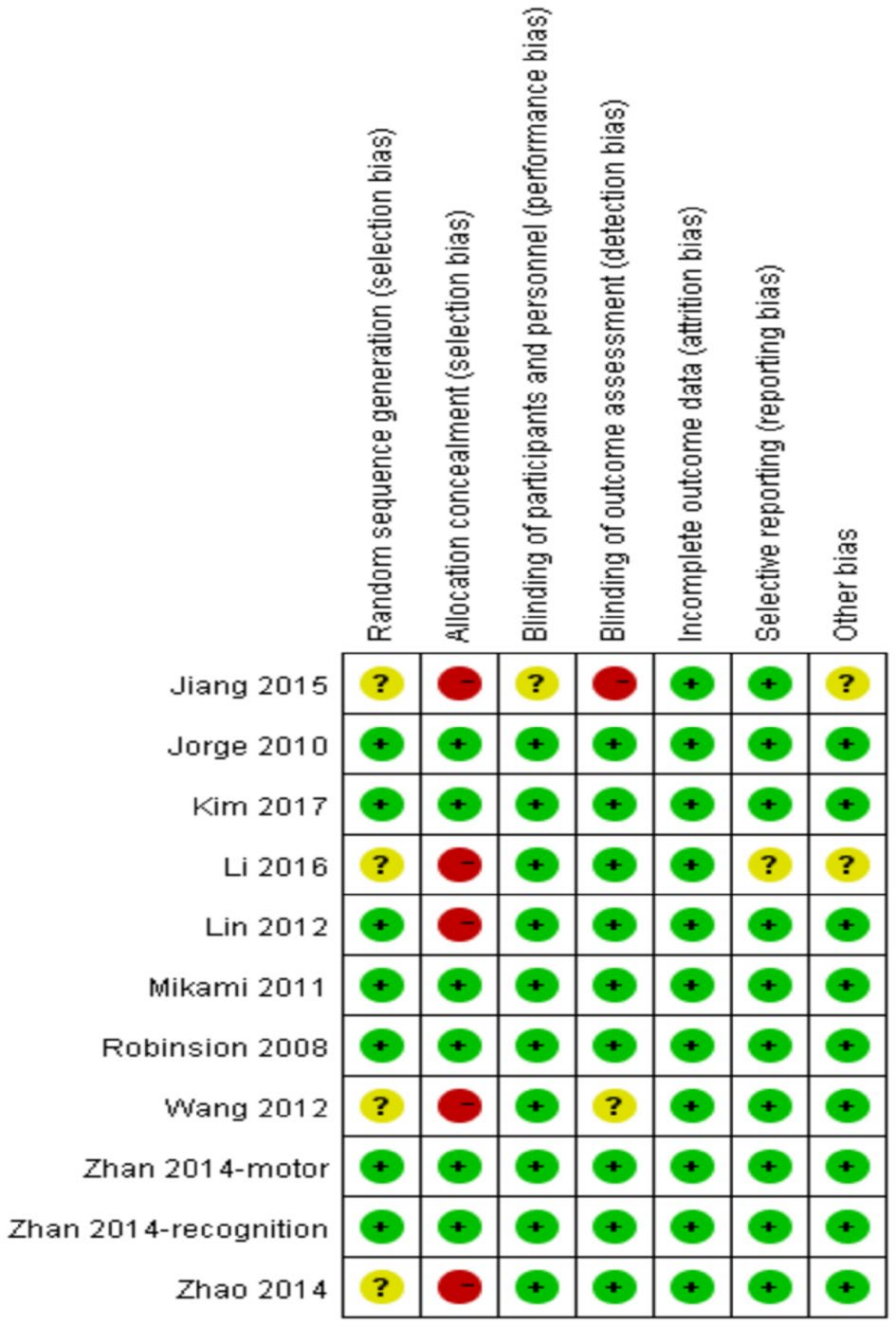
Review authors' judgments about each risk of bias item for each included study.

**Figure 9:**
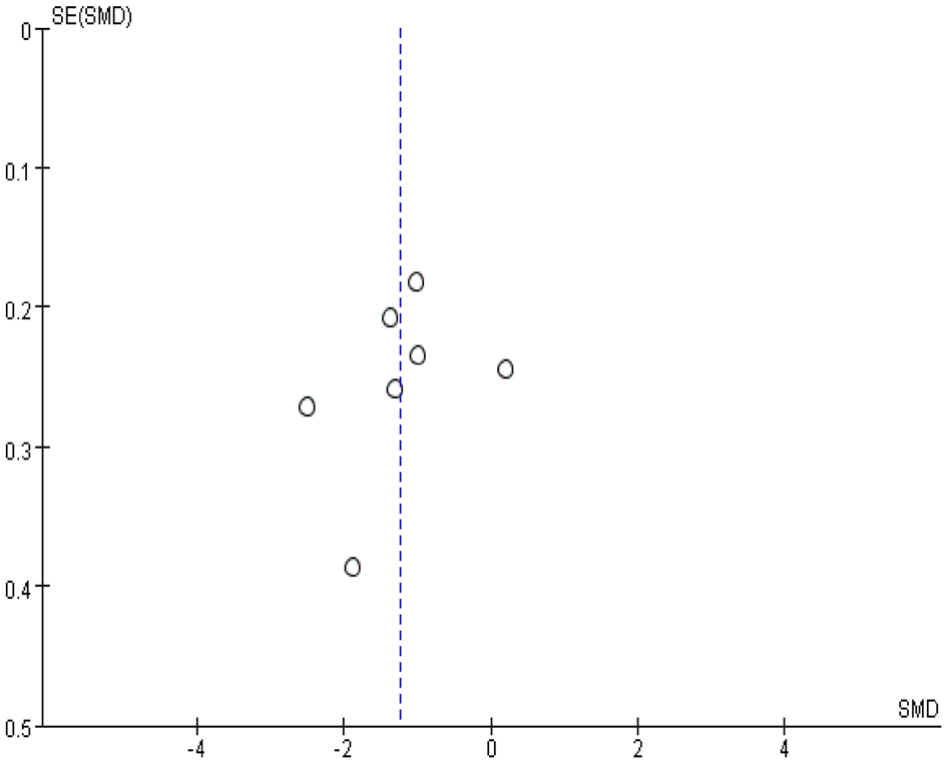
Invert funnel plots of the depression rating scores.

### Neurological deficit scores

The SMD was − 0.97 (95% CI, − 1.97 to 0.03; 4 trials; Supplemental Fig. 16) with high heterogeneity among trials (I^2^ = 97%), regarding different scales NFI (Neurologic Function Impairment) (SMD = -3.25; 95% CI, − 3.86 to − 2.64) vs NIHSS (SMD = -0.15; 95% CI, − 0.46 to 0.17; I^2^ = 91%, *P* = 0.15) vs MESSS (SMD = -0.35; 95% CI, − 0.69 to − 0.01). It was reported that the recovery rate of neurological function was obvious statistical significance (*P* < 0.05) in escitalopram group (86.1%) compared with the control (58.8%) in one trail, but we could not obtain the detailed scores^23^.

### Activities of daily living

The pooled analysis was not in favor of the escitalopram compared with the control (SMD = 0.42; 95% CI, − 0.32 to 1.16; I^2^ = 94%; Supplemental Fig. 17).

### Cognitive impairments

The SMD was 0.56 (95% CI, − 0.23 to 1.34; 3 trials; Supplemental Fig. 18) with high heterogeneity among trials (I^2^ = 94%; *P* < 0.001).

### Motor function

There was a better effect in the escitalopram versus the control (SMD = 0.47; 95% CI, 0.02 to 0.93; 4 trials, Supplemental Fig. 19) with high heterogeneity among trials (I^2^ = 83%; *P* = 0.0005), between different motor function scales FM (SMD = 0.65; 95% CI, 0.25 to 1.06; I^2^ = 54%, *P* = 0.11) vs Hemispheric Stroke Scale (SMD = 0.00; 95% CI, − 0.18 to 0.18).

### Quality assessment and sensitivity analyses

The quality of studies enrolled was shown in Fig. 8. In the sensitivity analyses, studies of the low quality were eliminated^19,20,22^, and the conclusions of pooled analyses were robust, except for the motor function (SMD = 0.36; 95% CI, − 0.40 to 1.13; I^2^ = 90%, *P* = 0.002) and the drowsiness (SMD = 4.70; 95% CI, 0.17 to 127.25; I^2^ = 64%, *P* = 0.09). Moreover, the I^2^ was decreased from 94 to 79% in the pooled analysis of the ADL. The inverted funnel plot of visual examination depression score (Fig. 9) was symmetrical. Moreover, the Egger tests showed that the outcome of depression rating scores (*t* = -0.77; *P* = 0.478 > 0.10) was not affected by publication bias.

## Discussion

The systematic review and meta-analysis give an up-to-date and detailed description of the efficacy of escitalopram for PSD, in which 11 papers and 1374 participants were enrolled. Excitingly, participants allocated to the escitalopram were more improved than the control, including depression rating scores, the incidence of PSD and motor function, but the participants enrolled in the escitalopram group did not experience more improved in aspect of the ADL, neurological function and cognitive function. Furthermore, the participants in the escitalopram groups did not suffer more adverse events compared with the control groups in our research, except for the drowsiness. However, in sensitivity analyses, the conclusions of motor function and the drowsiness were not stable, which should be considered carefully.

Our research reveals escitalopram reduces effectively the depression rating scores and the incidence of PSD, which demonstrates the escitalopram is effective in the treatment and prevention of PSD. Escitalopram is safe for stroke patients in our meta-analysis. The pooled results show the participants treated with escitalopram are well tolerated for adverse events, including the gastrointestinal, cardiovascular, sexual and other adverse events but the drowsiness which only 2 trails were enrolled, however, the escitalopram groups did not experience more drowsiness in the sensitivity analyses, which is consistent with our previous meta-analysis^26^ and is different from two previous meta-analyses^13,27^, due to different types of antidepressants enrolled in that researches, especially tricyclic antidepressants included in Xu et al.^27^. It is negative for the functional indexes we analyzed except for the motor function. In the sensitivity analyses, only the result of motor function is altered. The conclusions of functional indexes are not consistent with previous meta-analyses^26–28^, which maybe only ≤ 4 papers are enrolled in each functional index and the validity should be interpreted cautiously and proved in the future.

One of the potential weaknesses was the high heterogeneity among trials in our research, except for the incidence of PSD and adverse events. The possible reasons were analyzed. Firstly, it may be small samples of most trials we enrolled and low quality of some trails, which may lead to high risk of bias and overestimation^29^. So in the sensitivity analyses, the I^2^ was decreased from 94 to 79% in the pooled analysis of the ADL. Secondly, different rating scales were used in the studies included, and one study shows the occurrence of PSD is different by different depression scales (HAM-D_17_ vs. HAM-D_6_)^30^, which proved different scales could lead to different outcomes and conclusions. It is testified in the subgroup analyses of neurological function (Supplemental Fig. 16) and motor function (Supplemental Fig. 19). Thirdly, after removing the paper^16^, the I^2^ was decreased from 90 to 80% in the pooled analysis of the depression rating scores, and the reason maybe the intervention duration (12 months) was obviously longer than other trails enrolled. Fourthly, after removing the trail^12^, heterogeneity (I^2^ = 22%, *P* = 0.26) decreased significantly in the pooled analysis of cognitive function, which maybe the weight of the trail was too large and the studies enrolled were too few.

The main problems of the serious cognitive impairment, aphasia, and the severe stroke about the participants were excluded in the studies recruited, therefore we could not know whether those patients could be treated with escitalopram, which was also a limitation of our research. Another limitation was that the studies enrolled were too few in some pooled analyses and related original studies should be conducted to clarify and testify the results.

The strengths also existed in our study. An extensive search was conducted, including online papers, references and unpublished trials, as well as a fairly wide range of important clinical results. Additionally, sufficient sensitivity analyses and enough subgroup analyses were performed to ensure the reliability and robustness of the results. In the future, it should be necessary to develop more detailed and rigorous basic experiments on mechanisms and clinical trials to make a better choice for clinicians and patients.

## Conclusions

Taken together, our findings prompt escitalopram is safe and effective for PSD. However, the pooled analyses of the motor function and the incidence of drowsiness should be explained cautiously. Moreover, limitations and inspirations are provided for further researches in our study. Therefore, more multicenter, larger sample, more rigorous and more result indexes designed RCTs are needed to evaluate the protective role of escitalopram on PSD.

## Supplementary Information

Supplementary Information.
