# Publisher Correction: Efficacy of escitalopram for poststroke depression: a systematic review and meta-analysis

**DOI:** 10.1038/s41598-022-10039-9

**Published:** 2022-04-06

**Authors:** Rong-fang Feng, Rui Ma, Peng Wang, Xu Ji, Zhen-xiang Zhang, Meng-meng Li, Jia-wei Jiao, Li Guo

**Affiliations:** 1 The First Affiliated Hospital of Zhengzhou University, Zhengzhou, 450052 Henan People’s Republic of China; 2 College of Physical Education (Based School), Zhengzhou University, Zhengzhou, 450001 Henan People’s Republic of China; 3 Department of Basic Medicine, School of Nursing and Health, Zhengzhou University, Zhengzhou, 450001 Henan People’s Republic of China; 4 Zhengzhou University of Industrial Technology, Zhengzhou, 450002 Henan People’s Republic of China; 5 Medical School of Huanghe Science and Technology University, Zhengzhou, 450006 Henan People’s Republic of China; 6 Henan University of Chinese Medicine, Zhengzhou, 450046 Henan People’s Republic of China; 7 Department of Clinical Medicine, School of Nursing and Health, Zhengzhou University, Zhengzhou, 450001 Henan People’s Republic of China; 8 Zhengzhou Central Hospital Affiliated to Zhengzhou University, Zhengzhou, 450007 People’s Republic of China

Correction to: *Scientific Reports* https://doi.org/10.1038/s41598-022-05560-w, published online 28 February 2022

The original version of the Article contained an error in the Author list, where the authors Rong‑fang Feng and Rui Ma were erroneously listed as equally contributing authors.

The original Article has been corrected.

