# Supplementary material for: Efficacy of escitalopram for poststroke depression: a systematic review and meta-analysis
Source: Sci Rep. 2022 Feb 28;12:3304. doi: 10.1038/s41598-022-05560-w (PMC8885641; doi:10.1038/s41598-022-05560-w)
Supplement: Supplementary file 1 — Supplementary Information. [file 41598_2022_5560_MOESM1_ESM.docx]

***Supplemental Material***

***Search strategies***

#1 randomized controlled trial [Publication Type]

#2 controlled clinical trial [Publication Type]

#3 randomized [Text Word]

#4 placebo[Text Word]

#5 drug therapy [MeSH Terms]

#6 randomly [Text Word]

#7 trial [Text Word]

#8 group[Text Word]

#9 OR/1-8

#10 humans [MeSH Terms]

#11 patients [MeSH Terms]

#12 participants[Text Word]

#13 OR/10-12

#14 Stroke [Mesh]

#15 “Cerebral Hemorrhage” [MeSH Terms]

#16 “Cerebral infarction” [MeSH Terms]

#17 Apoplexy[Text Word]

#18 “Brain vascular accident” [Text Word]

#19 “Cerebrovascular accident” [Text Word]

#20 “Cerebral stroke” [Text Word]

#21 “Brain infarction” [Text Word]

#22 “Intracranial hemorrhages” [Text Word]

#23 Hemiplegia [Text Word]

#24 OR/14-23

#25 Selective serotonin reuptake inhibitors [MeSH Terms]

#26 Antidepressive Agents [MeSH Terms]

#27 Antidepressive Agents, Second-Generation [MeSH Terms]

#28 Escitalopram[Text Word]

#29 OR/25-28

#30 depression [MeSH Terms]

#31 depressive disorder [MeSH Terms]

#32 OR/30, 31

#33 #9 AND #13 AND #24 AND #29 AND #32

***Supplemental Figure Legends***

Supplemental Figure 1............................................................Incidence of the dry mouth adverse events.

Supplemental Figure 2...............................................................Incidence of the anorexia adverse events.

Supplemental Figure 3...........................................................Incidence of the indigestion adverse events.

Supplemental Figure 4...............................................................Incidence of the bleeding adverse events.

Supplemental Figure 5...........................................................Incidence of the tachycardia adverse events.

Supplemental Figure 6.............................................................Incidence of the chest pain adverse events.

Supplemental Figure 7...........................................................Incidence of the drowsiness adverse events.

Supplemental Figure 8..............................................................Incidence of the insomnia adverse events.

Supplemental Figure 9..............................................................Incidence of the dizziness adverse events.

Supplemental Figure 10................................................................Incidence of the fatigue adverse events.

Supplemental Figure 11.............................................Incidence of the increased sweating adverse events.

Supplemental Figure 12....................................................................Incidence of the falls adverse events.

Supplemental Figure 13....................................................................Incidence of the pain adverse events.

Supplemental Figure 14...............................................................Incidence of the dysuria adverse events.

Supplemental Figure 15...............................................................Incidence of the anxiety adverse events.

Supplemental Figure 16.....................Neurological Deficit Scores of subgroup of different rating scales.

Supplemental Figure 17..........................................................................Activities of Daily Living scores.

Supplemental Figure 18..............................................................................Cognitive Impairments scores.

Supplemental Figure 19..............................Motor Function scores of subgroup of different rating scales.


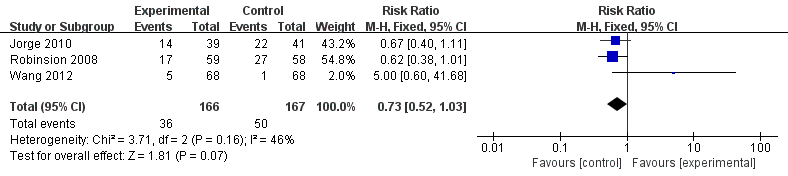


**Supplemental Figure 1.** Incidence of the dry mouth adverse events. CI, confidence interval.


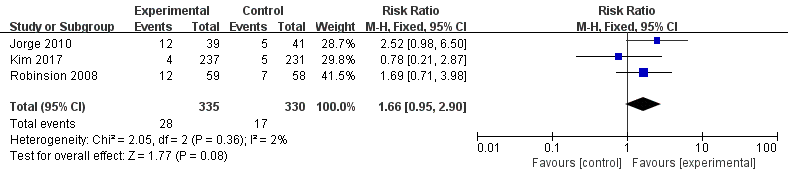


**Supplemental Figure 2.** Incidence of the anorexia adverse events. CI, confidence interval.


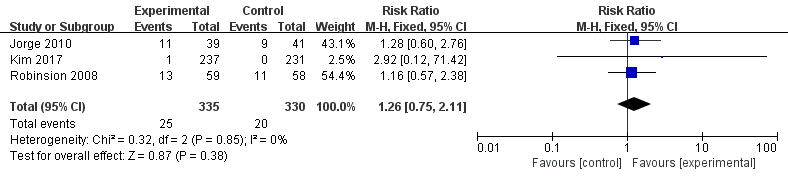


**Supplemental Figure 3.** Incidence of the indigestion adverse events. CI, confidence interval.


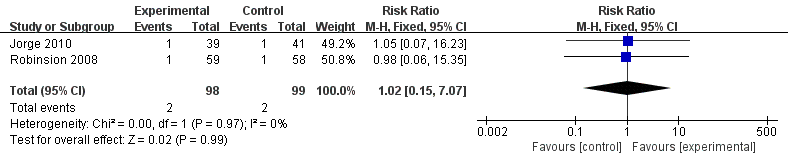


**Supplemental Figure 4.** Incidence of the bleeding adverse events. CI, confidence interval.


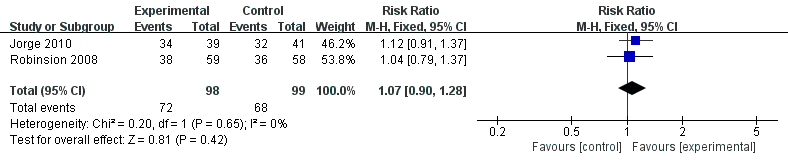


**Supplemental Figure 5.** Incidence of the tachycardia adverse events. CI, confidence interval.


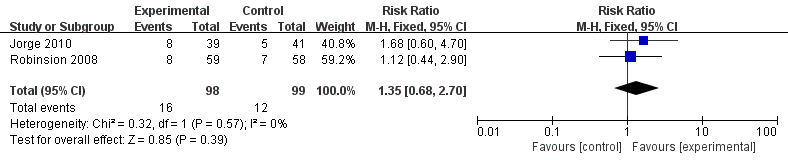


**Supplemental Figure 6.** Incidence of the chest pain adverse events. CI, confidence interval.


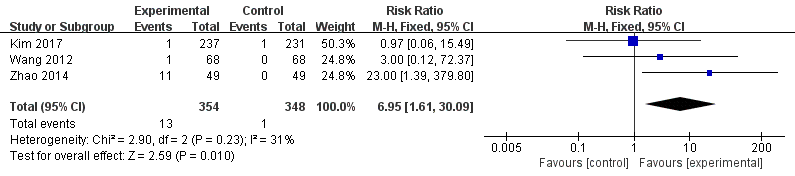


**Supplemental Figure 7.** Incidence of the drowsiness adverse events. CI, confidence interval.


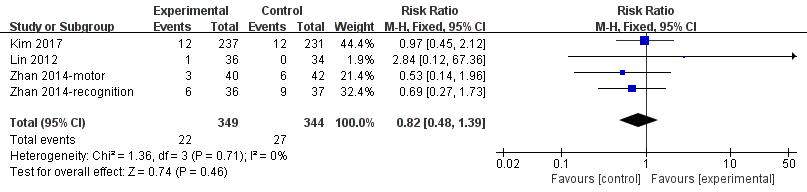


**Supplemental Figure 8.** Incidence of the insomnia adverse events. CI, confidence interval.


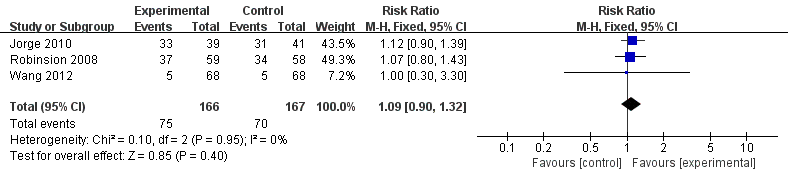


**Supplemental Figure 9.** Incidence of the dizziness adverse events. CI, confidence interval.


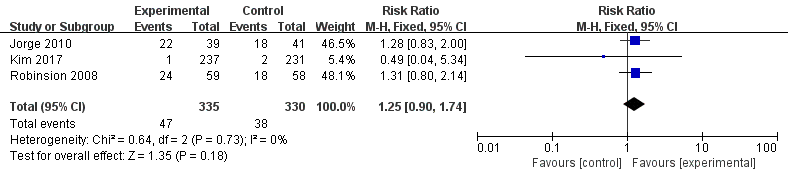


**Supplemental Figure 10.** Incidence of the fatigue adverse events. CI, confidence interval.


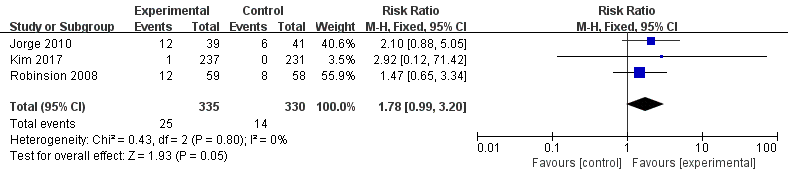


**Supplemental Figure 11.** Incidence of the increased sweating adverse events. CI, confidence interval.


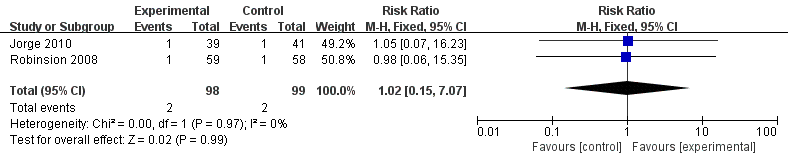


**Supplemental Figure 12.** Incidence of the falls adverse events. CI, confidence interval.


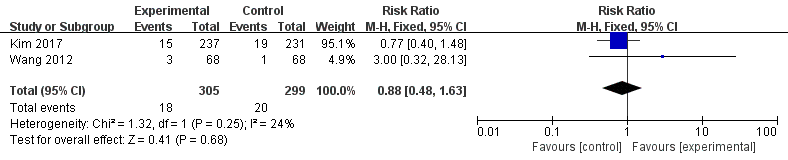


**Supplemental Figure 13.** Incidence of the pain adverse events. CI, confidence interval.


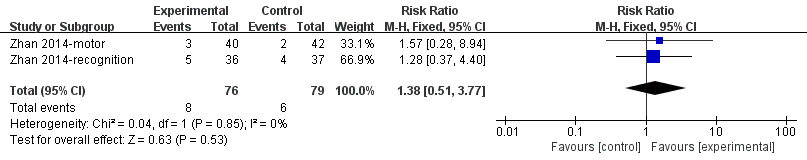


**Supplemental Figure 14.** Incidence of the dysuria adverse events. CI, confidence interval.


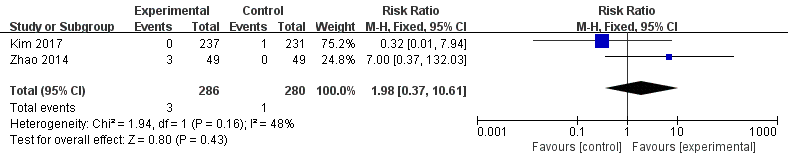


**Supplemental Figure 15.** Incidence of the anxiety adverse events. CI, confidence interval.


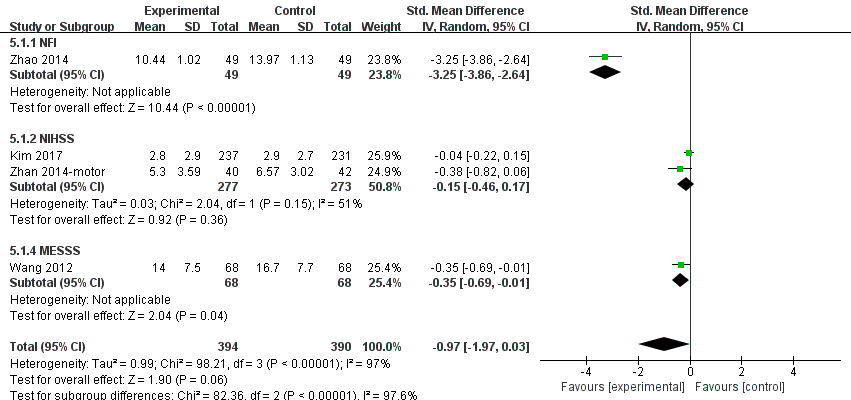


**Supplemental Figure 16.** Neurological Deficit Scores of subgroup of different rating scales. NFI, Neurologic Function Impairment; NIHSS, National Institutes of Health Stroke Scale; MESSS, Modified Edinburgh Scandinavia Stroke Scale. CI, confidence interval.


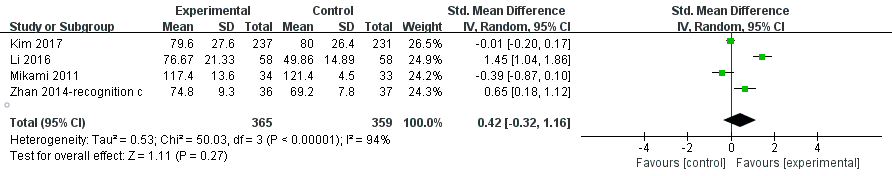


**Supplemental Figure 17.** Activities of Daily Living scores. CI, confidence interval.


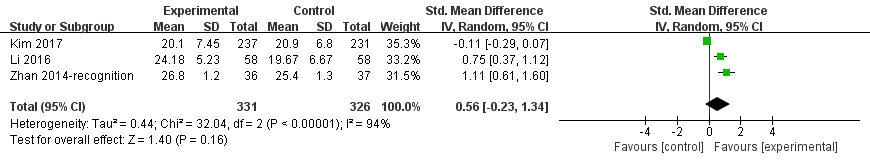


**Supplemental Figure 18.** Cognitive Impairments scores. CI, confidence interval.


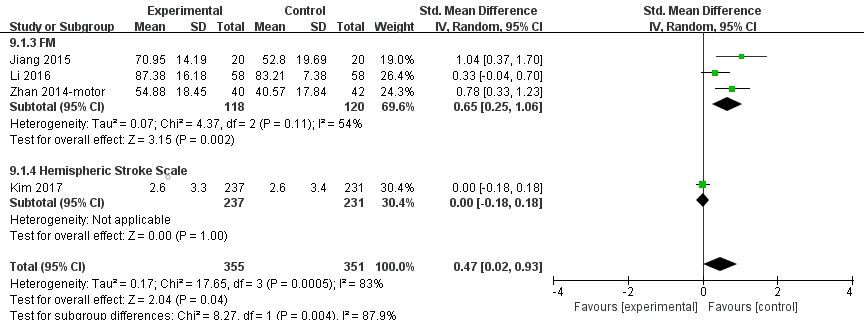


**Supplemental Figure 19.** Motor Function scores of subgroup of different rating scales. FM, Fugl-Meyer motor scale. CI, confidence interval.
